# Synthesis and biological evaluation of a new series of *ortho*-carboranyl biphenyloxime derivatives

**DOI:** 10.1186/s13065-018-0444-z

**Published:** 2018-06-29

**Authors:** Guofan Jin, Fuyan Xiao, Ruijiang Liu

**Affiliations:** School of Pharmacy, Jiangsu University, Zhenjiang, 212013 People’s Republic of China

**Keywords:** Carborane, Morpholine, Piperidine, HeLa cell, BPA

## Abstract

(*Z,Z’*)-1,1*′*-(4-*ortho*-Caboranyldimethyl)-bis(2-methoxyphenylethan-1-oxime) intermediate **3** was synthesized by a three-step reaction with a final treatment with base to give a new series of *ortho*-carboranyl biphenyloxime derivatives (**4**–**8**). Compounds **7** and **8** showed high solubility and the in vitro study results revealed high levels of accumulation in HeLa cells with higher cytotoxicity and boron uptake compared to l-boronphenylalanine.

**Electronic supplementary material:**

The online version of this article (10.1186/s13065-018-0444-z) contains supplementary material, which is available to authorized users.

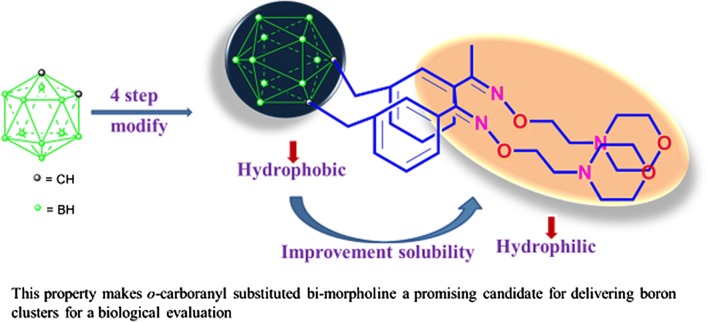

## Introduction

**Fig. 1:**
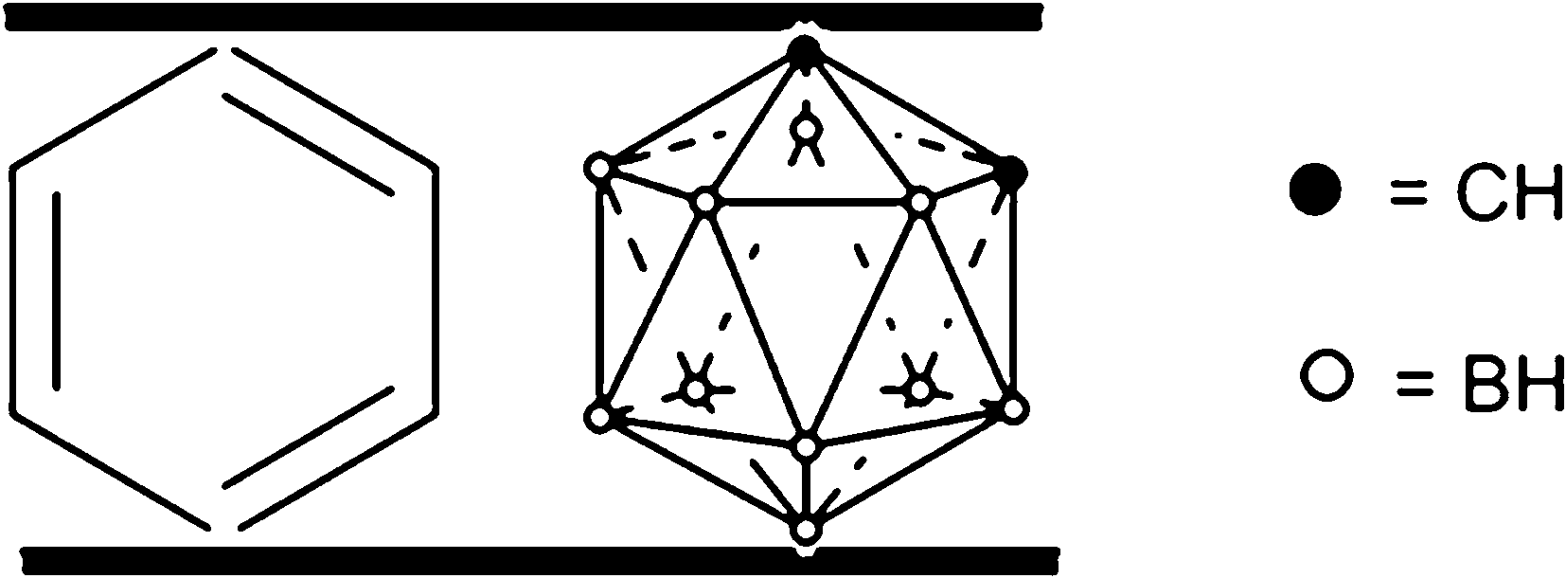
Comparison of the *o*-Carborane and benzene

Carborane (C_2_B_10_H_12_, Fig. 1) is a spherical compound formed by one or more boron peaks of polyhedral boron compounds, which is formed by carbon atoms. The volume is similar to that of a benzene ring [1–5]. This is a special large steric skeleton with a very strong hydrophobic structure. Therefore, improvement of the chemical structure can alter the stability, water solubility, and biological activity of compatibility and allow wider applications of carborane as a BNCT agent [6–9]. Boron neutron capture therapy (BNCT) was first proposed as a potential cancer therapy in 1936, based on the thermal neutron captured by ^10^B atoms then produces a ^4^He (α-particle) and a ^7^Li ion [10, 11]. However, its successful application in the treatment of cancer patients still presents a challenge in medical research [12]. A major challenge in designing boron containing drugs for BNCT of cancer is the selective delivery of ^10^B to the tumor as well as water solubility [13]. Our synthetic strategy was to use heterocyclic alkyl chains as a boron delivery system, the target molecules being the heterocyclic alkyl oxime chains in which the boron functionality was present as a *ortho*-carborane. The large number of boron atoms has a clear advantage for BNCT [14]. This paper reports the hydrophilic carboranylbenzyloxime moiety, such as alkylmorpholine, alkylpiperidine, phenoxyalkyl, and pyridine, on carbon–oxygen combined with chemical bonding. These compounds have higher solubility in polar solvents and increased the boron uptake in tumor cells, highlighting the potential use of carborane as a hydrophilic carrier into the body that can pass the Blood Brain Barrier (BBB rule) to the cells within the organization for drug evaluation.

## Experimental

All manipulations were performed under a dry nitrogen atmosphere using standard Schlenk techniques. Tetrahydrofuran (THF) was purchased from Aladdin Pure Chemical Company and dried over sodium metal distillation prior use. The reactions were monitored on Merck F-254 pre-coated TLC plastic sheets using hexane as the mobile phase. All yields refer to the isolated yields of the products after column chromatography using silica gel (200–230 mesh). All glassware, syringes, magnetic stirring bars, and needles were dried overnight in a convection oven. *Ortho*-carborane (C_2_H_2_B_10_H_10_) was purchased from HENAN WANXIANG Fine Chemical Company and used after sublimation. The NMR spectra were recorded on a Bruker 300 spectrometer operated and the chemical shifts were measured relative to the internal residual peaks from the lock solvent (99.9% CDCl_3_ and CD_3_COCD_3_), and then referenced to Si(CH_3_)_4_ (0.00 ppm). The Fourier transform infrared (FTIR) spectra of the samples were recorded on an Agilent Cary 600 Series FT-IR spectrometer using KBr disks. Elemental analyses were performed using a Carlo Erba Instruments CHNS–O EA1108 analyzer (Additional file 1).

### Synthetic routes and experimental data

Synthesis of bis(3-methoxybenzyl)-*ortho*-carborane (**1**). A 2.5 M *n*-BuLi (4.0 mL, 10 mmol) solution was added via a syringe to a solution of *o*-carborane (1.44 g, 10 mmol) in 50 mL of THF at − 78 °C. A solution of 1-(bromomethyl)-3-methoxybenzene (4.22 g, 21 mmol) in THF 10 mL was added slowly to the reaction flask at − 78 °C, and the reaction temperature was maintained at − 78 °C for 1 h. The reaction mixture was then warmed slowly to room temperature, stirred for an additional 12 h, and quenched with distilled H_2_O (30 mL). The crude product was then extracted with methylene chloride (30 mL × 3). The organic layer was washed with H_2_O, dried with anhydrous Na_2_SO_4_, and filtered then concentrated. The residue was purified by flash column chromatography (ethyl acetate/hexane 1:10) to give compound **1** as a colorless oil: yield: 3.6 g (93%). IR(KBr pellet), cm^−1^, ν: (B-H_*o*-carborane_) 2593. ^1^HNMR (CDCl_3_), δ, ppm: 3.2–0.8 (br, B-H_*o*-carborane_, 10H), 3.61 (s, –CH_2_, 4H), 3.83 (s, –OCH_3_, 6H), 6.77 (s, 1-H_benzene_, 2H), 6.84–6.82 (d, *J* = 6.9 Hz, 2-H_benzene_, 2H), 6.90–6.88 (d, *J* = 6.9 Hz, 3-H_benzene_, 2H), 7.32–7.29 (m, 4-H_benzene_, 2H). Found, %: C 56.31; H 7.65. C_18_H_28_B_10_O_2_. Calculated, %: C 56.23; H 7.34.

Synthesis of 1,1′-(4-caboranyldimethyl)-bis(2-methoxy-4,1-phenylene-ethan-1-one) (**2**). Acetyl chloride (1.4 mL, 20 mmol) was added via a syringe to a solution of aluminum chloride (2.6 g, 20 mmol) in 50 mL of methylene chloride at 0 °C and stirred for 30 min. A solution of compound **1** (3.5 g, 10 mmol) in methylene chloride 10 mL was added slowly to the reaction flask at 0 °C, and the reaction temperature was maintained at 0 °C for 30 min. The reaction mixture was then warmed slowly to room temperature, stirred for an additional 3 h, and quenched with a saturated NaHCO_3_ (30 mL) solution. The crude product was then extracted, and the organic layer was washed with H_2_O, dried with anhydrous Na_2_SO_4_, and filtered then concentrated. The residue was purified by flash column chromatography (ethyl acetate/hexane 1:8) to give compound **2** as a colorless oil: yield: 4.1 g (97%). IR (KBr pellet), cm^−1^, ν: (B-H_*o*-carborane_) 2602. ^1^HNMR(CDCl_3_), δ, ppm: 3.2–0.8 (br, B-H_*o*-carborane_, 10H), 3.64 (s, –CH_3_, 6H), 3.66 (s, –CH_2_, 4H), 3.95 (s, –OCH_3_, 6H), 6.82 (s, 1-H_benzene_, 2H), 6.89–6.86 (d, *J* = 7.8 Hz, 2-H_benzene_, 2H), 7.77–7.74 (d, *J *= 7.8 Hz, 3-H_benzene_, 2H). Found, %: C 56.42; H 6.67. C_22_H_32_B_10_O_4_. Calculated, %: C 56.39; H 6.88.

Synthesis of (*Z*,*Z*′)-1,1′-(4-caboranyldimethyl)-bis(2-methoxyphenylethan-1-oxime) (**3**). A solution of compound **2** (3.8 g, 8.1 mmol) and hydroxylamine (1.2 g, 17.8 mmol) in 40 mL of methanol was heated under reflux for 2 h. The reaction mixture was then cooled to room temperature, and the crude product was concentrated. The residue was purified by flash column chromatography (ethyl acetate/hexane 1:4) to give compound **3** as a colorless oil: Yield: 3.7 g (92%). IR (KBr pellet), cm^−1^, ν: (B-H_*o*-carborane_) 2586. ^1^H NMR (CD_3_COCD_3_), δ, ppm: 3.16 (s, –CH_3_, 6H), 3.2–0.8 (br, B-H_*o*-carborane_, 10H), 3.88 (s, –OCH_3_, 6H), 3.93 (s, –CH_2_, 4H), 6.97–6.95 (d, *J *= 7.5 Hz, 2-H_benzene_, 2H), 7.05 (s, 1-H_benzene_, 2H), 7.30–7.28 (d, *J* = 7.5 Hz, 3-H_benzene_, 2H). Found, %: C 52.68; H 6.81; N 5.69. C_22_H_34_B_10_N_2_O_4_. Calculated, %: C 52.99; H 6.87; N 5.62.

Synthesis of (1*Z,*1′*Z*)-1,1′-(carboranyldimethyl)-bis(2-methoxy-4,1-phenylene-ethan-1-one)-*O,O*-dipyridin-2-ylmethyldioxime (**4**). A solution of compound **3** (0.7 g, 1.4 mmol) and potassium carbonate (0.4 g, 3.0 mmol) in 10 mL of acetonitrile was stirred at room temperature for 30 min. Subsequently, (2-bromomethyl)pyridine (0.5 g, 3.0 mmol) was added at room temperature, and then heated under reflux for 5 h. The crude product was then concentrated, and the residue was purified by flash column chromatography (ethyl acetate/hexane 1:4) to give compound **4** as a yellow oil: Yield: 0.8 g (88%). IR (KBr pellet), cm^−1^, ν: (B-H_*o*-carborane_) 2607. ^1^HNMR (CD_3_Cl), δ, ppm: 2.31 (s, –CH_2_, 6H), 3.2–0.8 (br, B-H_*o*-carborane_, 10H), 3.63 (s, –CH_3_, 4H), 3.84 (s, –OCH_3_, 6H), 5.37 (s, –CH_2_, 2H), 6.73 (s, 1-H_benzene_, 2H), 6.80–6.77 (d, *J *= 7.8 Hz, 2-H_benzene_, 2H), 7.29–7.24 (m, 3-H_benzene and pyridine_, 4H), 7.47–7.44 (d, *J *= 7.8 Hz, 3-H_pyridine_, 2H), 7.76–7.70 (t, *J *= 7.8 Hz, 2-H_pyridine_, 2H), 8.61–8.59 (d, *J *= 4.8 Hz, 1-H_pyridine_, 2H). Found, %: C 59.36; H 6.63; N 8.35. C_34_H_44_B_10_N_4_O_4_. Calculated, %: C 59.98; H 6.51; N 8.23.

Synthesis of (1*Z,*1′*Z*)-1,1′-(carboranyldimethyl)-bis(2-methoxy-4,1-phenylene-ethan-1-one)-*O,O*-di(2-phenoxyethyl)dioxime (**5**). A procedure analogous to the preparation of **4** was used and a colorless oil was obtained. Yield: 0.9 g (89%). IR (KBr pellet), cm^−1^, ν: (B-H_*o*-carborane_) 2577. ^1^H NMR (CD_3_Cl) δ, ppm: 2.22 (s, –CH_3_, 6H), 3.2–0.8 (br, B-H_*o*-carborane_, 10H), 3.64 (s, –CH_2_, 4H), 3.85 (s, –OCH_3_, 6H), 4.31–4.28 (t, *J *= 4.8 Hz, –CH_2 alkyl-1_, 4H), 4.56–4.52 (t, *J *= 5.1 Hz, –CH_2 alkyl-2_ 4H), 6.75 (s, 1-H_benzene-1_ 2H), 6.83–6.80 (d, *J *= 7.5 Hz, 2-H_benzene-1_, 2H), 7.00–6.95 (m, 1-H_benzene-2_, 6H), 7.34–7.29 (m, 2-H_benzene-1 and 2_, 6H). Found, %: C 61.47; H 6.92; N 3.84. C_38_H_50_B_10_N_2_O_6_. Calculated, %: C 61.77; H 6.82; N 3.79.

Synthesis of (1*Z,*1′*Z*)-1,1′-(carboranyldimethyl)-bis(2-methoxy-4,1-phenylene-ethan-1-one)-*O,O*-di(3-phenoxypropyl)dioxime (**6**). A procedure analogous to the preparation of **4** was used and a colorless oil was obtained. Yield: 0.9 g (86%). IR (KBr pellet), cm^−1^, ν: (B–H) 2589. ^1^H NMR(CD_3_Cl), δ, ppm: 2.25–2.17 (m, –CH_3_ and -CH_2 alkyl-1_, 10H), 3.2–0.8 (br, B-H_*o*-carborane_, 10H), 3.64 (s, –CH_2_, 4H), 3.85 (s, –OCH_3_, 6H), 4.16–4.12 (t, *J *= 6.0 Hz, –CH_2 alkyl-2_, 4H), 4.40–4.36 (t, *J *= 6.0 Hz, –CH_2 alkyl-3_, 4H), 6.74 (s, 1-H_benzene-1_, 2H), 6.82–6.79 (d, *J *= 7.8 Hz, 2-H_benzene-1_, 2H), 6.96–6.93 (m, 1-H_benzene-2_, 6H), 7.33–7.30 (m, 2-H_benzene-1 and 2_, 6H). Found, %: C 62.52; H 7.12; N 3.77. C_40_H_54_B_10_N_2_O_6_. Calculated, %: C 62.64; H 7.10; N 3.65.

Synthesis of (1*Z,*1′*Z*)-1,1′-(carboranyldimethyl)-bis(2-methoxy-4,1-phenylene-ethan-1-one)-*O,O*-di(2-piperidin-1-ylethyl)dioxime (**7**). A procedure analogous to the preparation of **4** was used and a colorless oil was obtained. Yield: 0.8 g (82%) colorless oil. IR (KBr pellet), cm^−1^, ν: (B-H_*o*-carborane_) 2591. ^1^H NMR (CD_3_Cl), δ, ppm: 1.47–1.45 (m, 1-H_piperidine_, 4H), 1.64–1.60 (m, 2-H_piperidine_, 4H), 1.88–1.86 (m, 3-H_piperidine_, 4H), 2.19 (s, –CH_3_, 6H), 2.53–2.51 (m, 8H), 2.76–2.72 (t, *J *= 6.0 Hz, –CH_2 alkyl-1_, 4H), 3.2–0.8 (br, B-H_*o*-carborane_, 10H), 3.63 (s, –CH_2_, 4H), 3.85 (s, –OCH_3_, 6H), 4.36–4.32 (t, *J *= 6.0 Hz, –CH_2 alkyl-2_, 4H), 6.74 (s, 1-H_benzene_, 2H), 6.82–6.79 (d, *J *= 7.8 Hz, 2-H_benzene_, 2H), 7.31–7.29 (d, *J *= 7.8 Hz, 3-H_benzene_, 2H). Found, %: C 59.65; H 8.34; N 7.68. C_36_H_60_B_10_N_4_O_4_. C 59.97; H 8.39; N 7.77.

Synthesis of (1*Z,*1′*Z)*-1,1′-(carboranyldimethyl)-bis(2-methoxy-4,1-phenylene-ethan-1-one)-*O,O*-di(2-morpholinoethyl)dioxime (**8**). A procedure analogous to the preparation of **4** was used and a colorless oil was obtained. Yield: 0.9 g (84%). IR (KBr pellet), cm^−1^, ν: (B-H_*o*-carborane_) 2596. ^1^HNMR (CD_3_Cl), δ, ppm: 2.52 (s, –CH_3_, 6H), 2.55–2.54 (m, –CH_2 alkyl-1_, 4H), 2.77–2.72 (t, *J *= 6.9 Hz, –CH_2 alkyl-2_, 4H), 3.2–0.8 (br, B-H_*o*-carborane_, 10H), 3.64–3.59 (m, 1-H_morpholine_, 8H), 3.76–3.73 (m, 2-H_morpholine_, 8H), 3.85 (s, –OCH_3_, 6H), 6.83–6.76 (m, 2-H_benzene_, 4H), 7.31 (s, 2-H_benzene_, 2H). Found, %: C 56.38; H 7.83; N 7.64. C_34_H_56_B_10_N_4_O_6_. C 56.33; H 7.79; N 7.73.

### Cell viability assay (MTT assay)

HeLa cells in a 3 × 10^4^/mL cell suspension per hole in 96 well plates were digested by adding 100 μL of a cell suspension and culturing for 24 h to absorb the original culture medium followed by the addition of 200 μL configured compounds-**4**, **5**, **6**, **7**, **8** and BPA (l-boronphenylalanine). Each concentration was made from 4 compound holes, and the holes around the 96 well plates were sealed with PBS, the negative control. The blank control group lacked the compounds. After 24 h, 20 μL of a MTT solution was added to each hole, and cultured for 4 h. Subsequently, DMSO 150 μL was added to the medium through a suction hole and shaken for 10 min. The OD of each hole was determined at 490 nM, and the sample inhibition rate in different concentrations was calculated: inhibition rate = (Control OD value/Delivery OD value)/Control OD value × 100%. Finally, the IC_50_ value of the sample was calculated using the related software.

### Boron uptake

HeLa cells (5 × 10^3^) were incubated for 48 h in the presence of various concentrations of compounds **4**, **5**, **6**, **7**, **8**, and BPA. After washing three times, the cumulative boron concentration was determined by inductively coupled plasma atomic emission spectrometry (ICP-AES) [15, 16]. (± is the average value).

## Results and discussion

This paper reports the hydrophilic function of the *ortho*-carboranylbenzyloxime moiety, such as alkylmorpholine, alkylpiperidine, phenoxyalkyl and pyridine, on carbon–oxygen combined with chemical bonding. These compounds have higher solubility in polar solvents and increasing boron uptake in tumor cells within the organization for a drug evaluation.

**Scheme 1:**
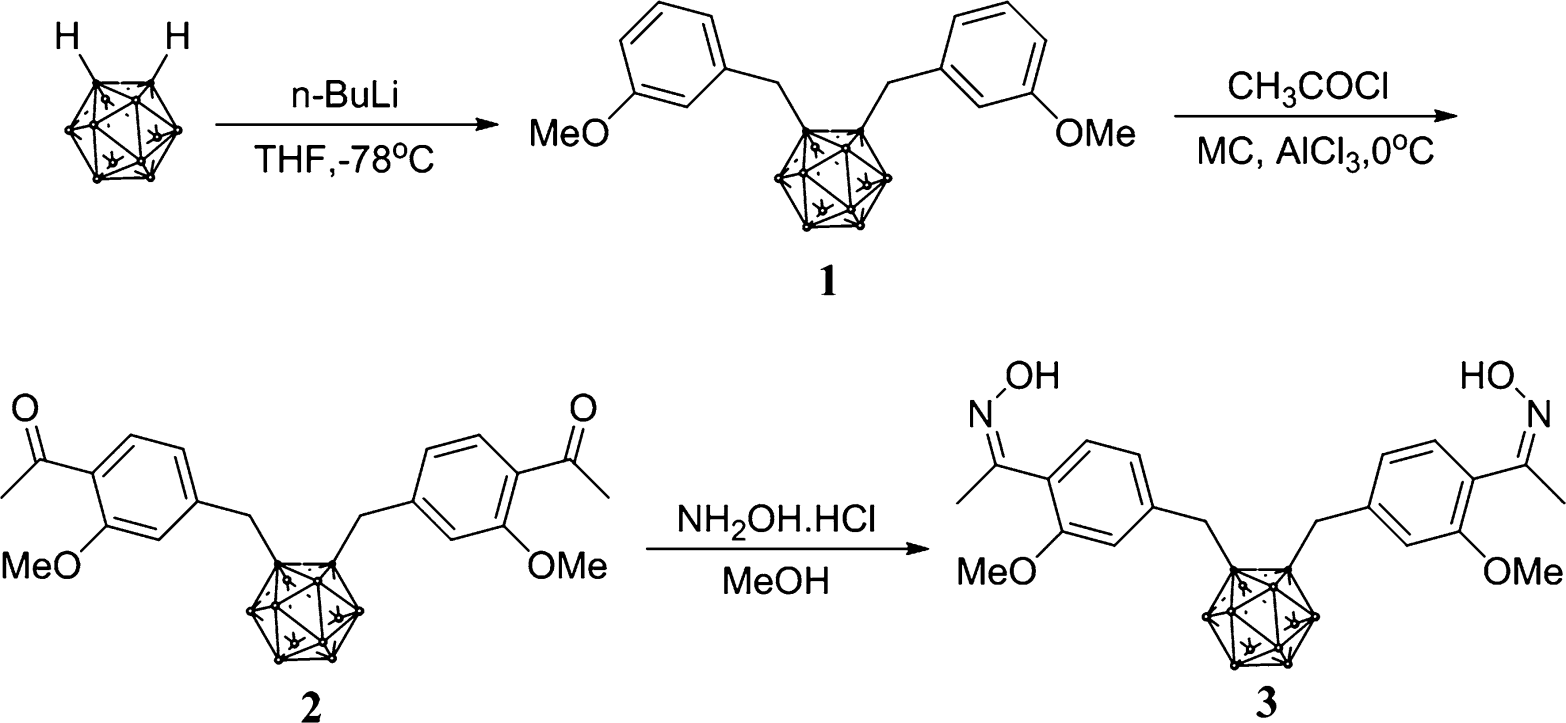
Preparation of (*Z,Z’*)-1, 1*′*-(4-Caboranyldimethyl)-bis(2-methoxyphenylethan-1-oxime)

A general procedure for the preparation for 4-*ortho*-caboranyldimethyl-bis(phenyloxime) consisted of a serial reaction, such as Grignard, Friedel–Crafts, amination, and electrophilic substitution under basic conditions. A series of carborane intermediates 1–3 were prepared using the optimized procedure from the starting material. *Ortho*-Carborane was dissolved in dry tetrahydrofuran at − 78 °C, and treated with a Grignard reagent carbanion, and then substituted with an aromatic halide. Subsequently, aluminum chloride was used in the Friedel–Craft reaction to afford 1,1′-(4-*ortho*-caboranyldimethyl)-bis(2-methoxy-4,1-phenylene-ethan-1-one), which was followed by the addition of hydroxylamine-hydrochloride salt to give the (Z,Z′)-1,1′-(4-*ortho*-Caboranyldimethyl)-bis(2-methoxyphenylethan-1-oxime) form in the presence of compound-**3** (Scheme 1) [17–21].

**Scheme 2:**
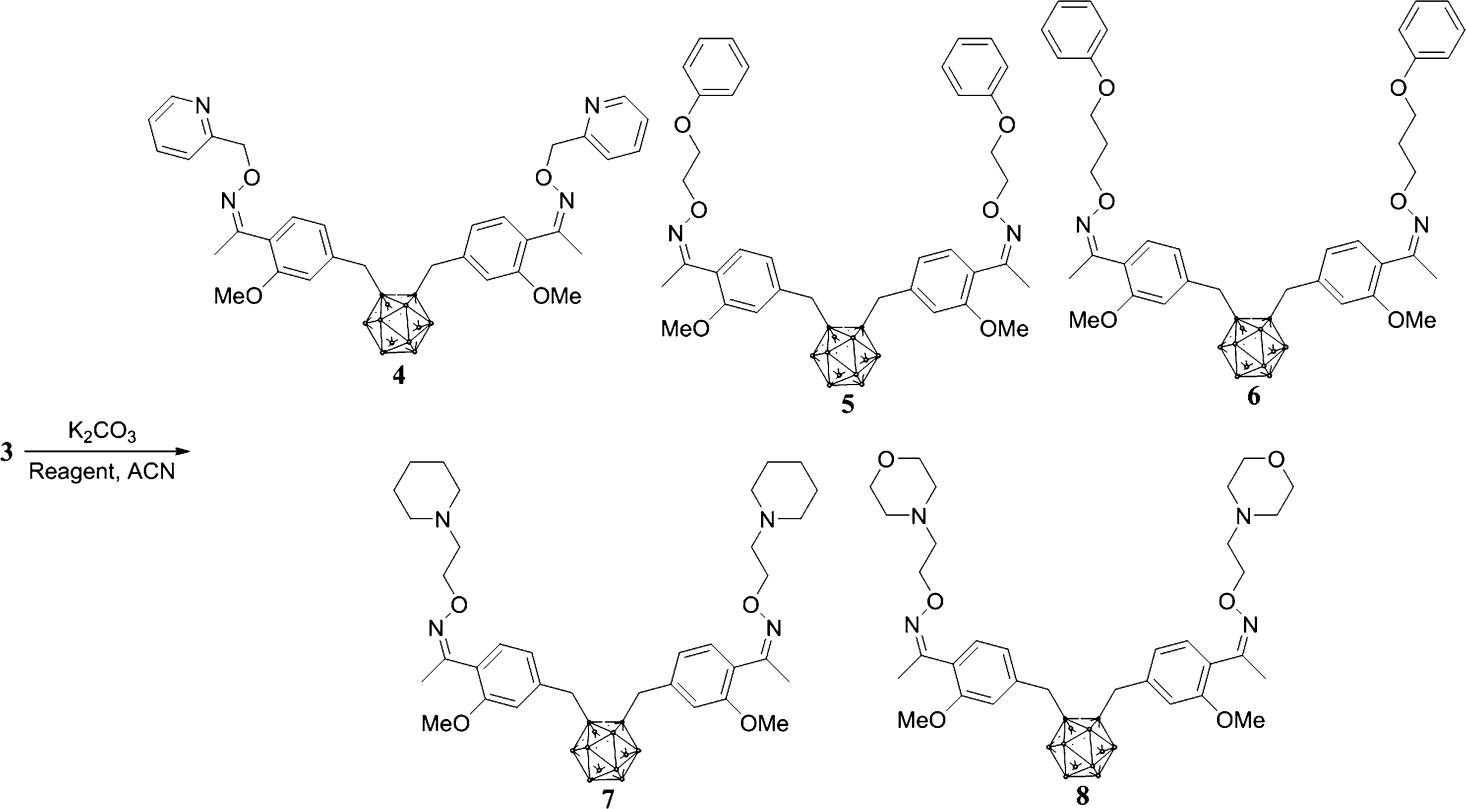
Preparation of (*Z,Z*′)-1,1*′*-(4-Caboranyldimethyl)-bis (hydrophilic functional) derivatives(**4**–**8**)

Finally, *ortho*-carboranyl hydrophilic ether compounds were generated from (*Z,Z*′)-1,1′-(4-*ortho*-Caboranyldimethyl)-bis(2-methoxyphenylethan-1-oxime) and side hydrophilic alkyl or aromatic halide reagents, followed by a treatment with potassium carbonate to result in the target compounds **4**–**8** (Scheme 2) [22, 23]. A treatment of *ortho*-carborane (C_2_H_2_B_10_H_10_) with aromatic halide as a base in tetrahydrofuran produced the target compounds **1**–**3** in moderate yields (1 93, 2 97, and 3 92%). Compounds **1**–**3** showed absorption bands in the infrared (IR) spectrum at 2602 and 2593 cm^−1^. The diagnostic signals of compounds **1**–**3** were the aromatic peaks observed at δ 7.77 and 6.77 in the ^1^H NMR spectra and a broad signal caused by B–H peaks for the *ortho*-carborane units from δ 3.2–0.8.

**Fig. 2:**
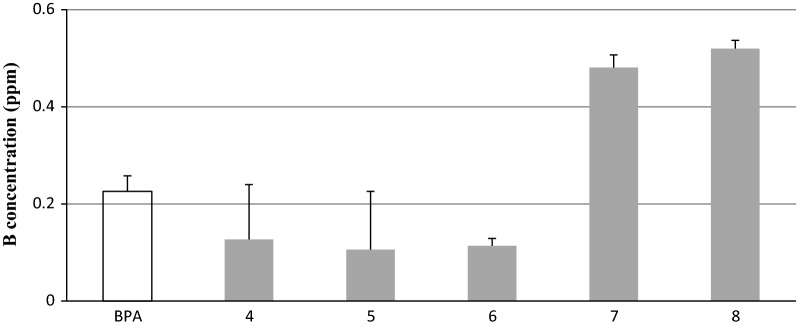
Accumulation of compounds **4**–**8** into HeLa cells

The major requirement of a BNCT agent is a high water solubility, high boron uptake, and low cytotoxicity. The HeLa cervical carcinoma cells were treated with the candidate compounds **4**–**8** for 2 days, and the cell viability was determined by a MTT assay. Compounds **4**–**8** exhibited boron uptake in the range of 0.106–0.520 ppm (Table 1), and the cell cytotoxicity was in the range of 1.134–2.516 µM, as shown Fig. 2. In particular, compounds **7** and **8** showed high boron uptake in HeLa cells, and both compounds had higher cytotoxicity than BPA (l-boronphenylalanine). Morpholine and piperidine is a heterocyclic nitrogen and oxygen member six-ring reagent with a simple structure that improves the water solubility and bioactivity improvement. They are used in the preparation of pharmaceutical drugs for their anti-inflammation, anticancer, and antiviral activity [24–28].

**Table 1:** Cytotoxicity (IC_50_) to HeLa cervical carcinoma cells

Compounds	Cytotoxicity IC_50_ (μM)^a^	Boron uptake (ppm)
**4**	2.516 ± 0.022	0.127 ± 0.113
**5**	1.924 ± 0.014	0.106 ± 0.120
**6**	2.383 ± 0.301	0.114 ± 0.015
**7**	1.582 ± 0.027	0.481 ± 0.026
**8**	1.134 ± 0.035	0.520 ± 0.017
BPA	4.16 ± 0.021	0.226 ± 0.016

^a^The results represent the means ± s.d.

## Conclusion

In conclusion, we reported the series of *ortho*-carborane substituted bipolar-function derivatives, such as alkyl pyridine, alkyl phenoxide, alkyl morpholine, and alkyl piperidine, were synthesized. The target compounds coupling of the aryl-oxime with chain functional group proceeded successfully for introduction of an *ortho*-carborane moiety in the molecules, which can easily be further four-step substituted to high yield final compound. The effects of synthesized compounds on biology activity were assay in HeLa cells. Both cyclic alkyl derivatives of *ortho*-carborane and oxime containing compounds, **7** and **8**, respectively, were exhibit high boron uptake and higher cytotoxicity than BPA (l-boronphenylalanine). This resulted in carborane compounds with improved water solubility for the BNCT agent. The knowledge gained from modified bipolar groups could facilitate both drug selection and evaluations.

## Additional file

**Additional file 1: Figure S1.** ^1^H-NMR bis(3-methoxybenzyl)carborane (**1**). **Figure S2.** ^1^H-NMR1,1′-(4-caboranyldimethyl)-bis(2-methoxy-4,1-phenylene-ethan-1-one) (**2**). **Figure S3.** ^1^H-NMR (*Z,Z*′)-1,1′-(4-caboranyldimethyl)-bis(2-methoxyphenylethan-1-oxime) (**3**). **Figure S4.** ^1^H-NMR (1*Z*,1′*Z*)-1,1′-(carboranyldimethyl)-bis(2-methoxy-4,1-phenylene-ethan-1-one)-*O,O*-dipyridin-2-ylmethyldioxime (**4**). **Figure S5.** ^1^H-NMR (1*Z*,1′*Z*)-1,1′-(carboranyldimethyl)-bis(2-methoxy-4,1-phenylene-ethan-1-one)-*O,O*-di(2-phenoxyethyl)dioxime (**5**). **Figure S6.** ^1^H-NMR (1*Z*,1′*Z*)-1,1’-(carboranyldimethyl)-bis(2-methoxy-4,1-phenylene-ethan-1-one)-*O,O*-di(3-phenoxypropyl)dioxime (**6**). **Figure S7.** ^1^H-NMR (1*Z*,1′*Z*)-1,1’-(carboranyldimethyl)-bis(2-methoxy-4,1-phenylene-ethan-1-one)-*O,O*-di(2-piperidin-1-ylethyl)dioxime (**7**). **Figure S8.** ^1^H-NMR (1*Z*,1′*Z)*-1,1’-(carboranyldimethyl)-bis(2-methoxy-4,1-phenylene-ethan-1-one)-*O,O*-di(2-morpholinoethyl)dioxime (**8**).
