# Supplementary material for: Synthesis and biological evaluation of a new series of ortho-carboranyl biphenyloxime derivatives
Source: Chem Cent J. 2018 Jun 29;12:76. doi: 10.1186/s13065-018-0444-z (PMC6026111; doi:10.1186/s13065-018-0444-z)
Supplement: Supplementary file 1 — Additional file 1: Figure S1. 1H-NMR bis(3-methoxybenzyl)carborane (1). Figure S2. 1H-NMR1,1′-(4-caboranyldimethyl)-bis(2-methoxy-4,1-phenylene-ethan-1-one) (2). Figure S3. 1H-NMR (Z,Z′)-1,1′-(4-caboranyldimethyl)-bis(2-methoxyphenylethan-1-oxime) (3). Figure S4. 1H-NMR (1Z,1′Z)-1,1′-(carboranyldimethyl)-bis(2-methoxy-4,1-phenylene-ethan-1-one)-O,O-dipyridin-2-ylmethyldioxime (4). Figure S5. 1H-NMR (1Z,1′Z)-1,1′-(carboranyldimethyl)-bis(2-methoxy-4,1-phenylene-ethan-1-one)-O,O-di(2-phenoxyethyl)dioxime (5). Figure S6. 1H-NMR (1Z,1′Z)-1,1’-(carboranyldimethyl)-bis(2-methoxy-4,1-phenylene-ethan-1-one)-O,O-di(3-phenoxypropyl)dioxime (6). Figure S7. 1H-NMR (1Z,1′Z)-1,1’-(carboranyldimethyl)-bis(2-methoxy-4,1-phenylene-ethan-1-one)-O,O-di(2-piperidin-1-ylethyl)dioxime (7). Figure S8. 1H-NMR (1Z,1′Z)-1,1’-(carboranyldimethyl)-bis(2-methoxy-4,1-phenylene-ethan-1-one)-O,O-di(2-morpholinoethyl)dioxime (8). [file 13065_2018_444_MOESM1_ESM.docx]

Additional information

Synthesis and biological evaluation of a new series of *ortho*-carboranyl biphenyloxime derivatives

Guofan, Jin^*^ Fuyan Xiao and Ruijiang Liu

School of Pharmacy, Jiangsu University, Zhenjiang, 212013, P. R. China

^*^E-mail: [organicboron@ujs.edu.cn](mailto:organicboron@ujs.edu.cn)


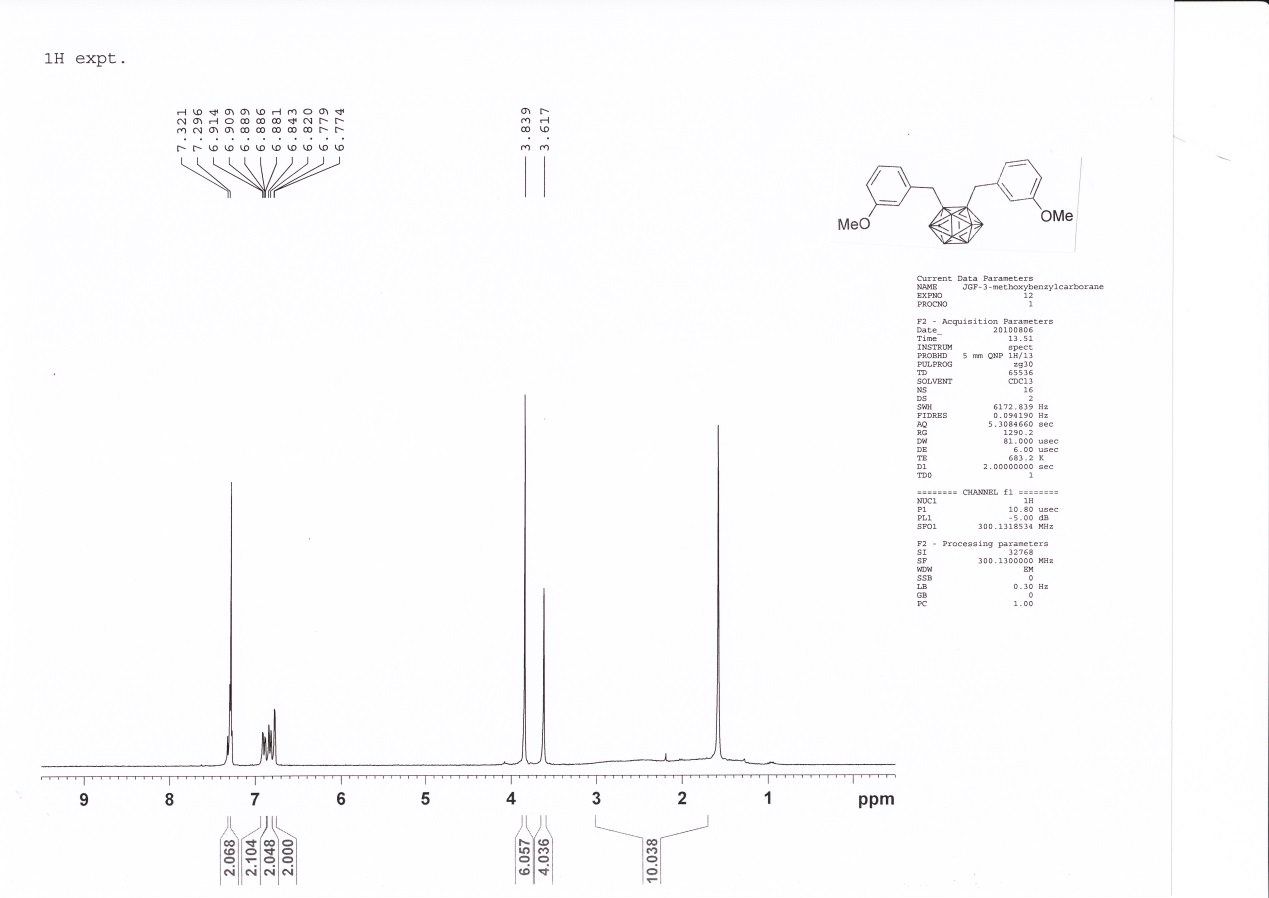


Additional file 1: Fig S1. ^1^H-NMR bis(3-methoxybenzyl)carborane (**1**)


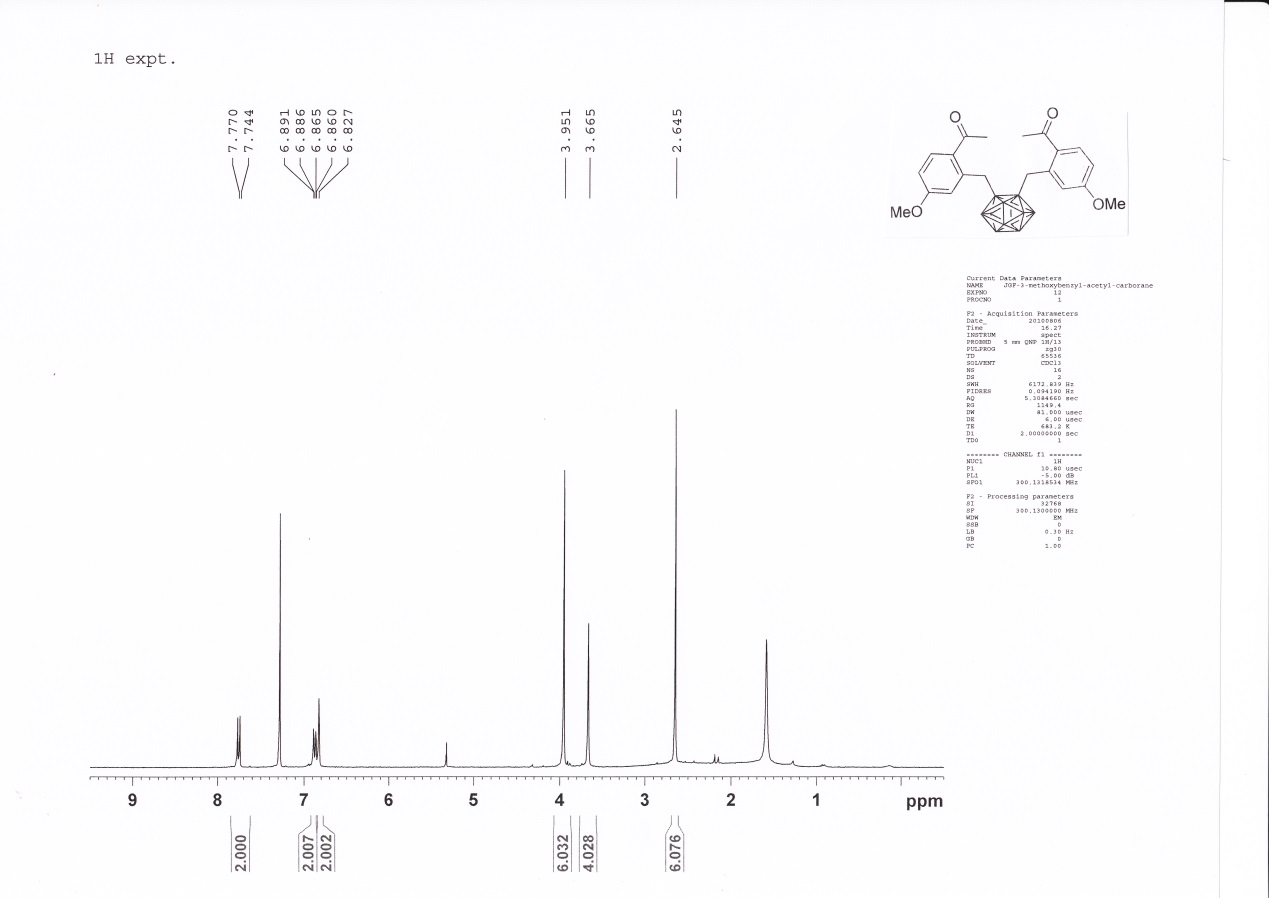


Additional file 1: Fig S2. ^1^H-NMR1,1'-(4-caboranyldimethyl)-bis(2-methoxy-4,1-phenylene-ethan-1-one) (**2**)


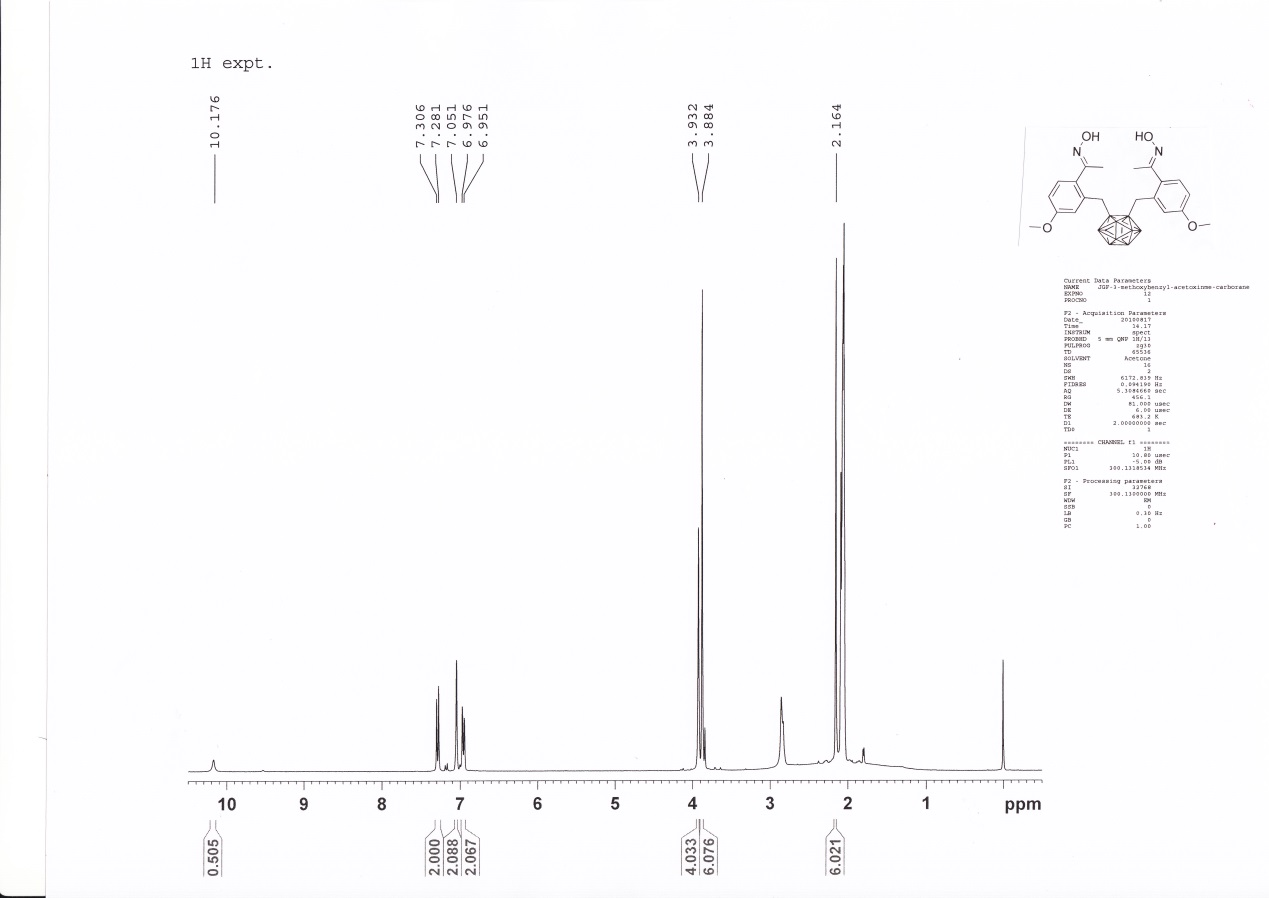


Additional file 1: Fig S3. ^1^H-NMR (*Z,Z'*)-1,1'-(4-caboranyldimethyl)-bis(2-methoxyphenylethan-1-oxime) (**3**)


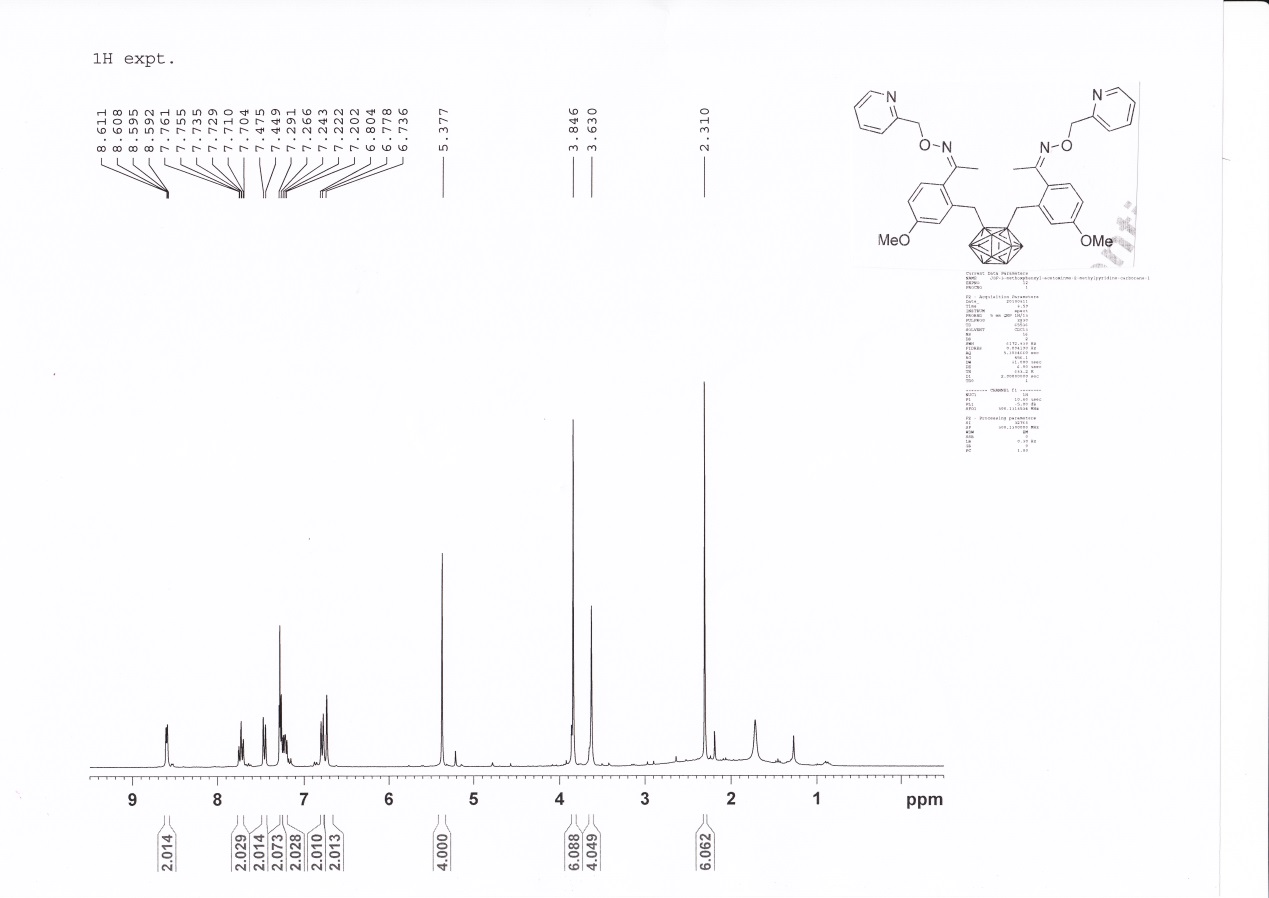


Additional file 1: FigS4. ^1^H-NMR (1*Z,*1*'Z*)-1,1'-(carboranyldimethyl)-bis(2-methoxy-4,1-phenylene-ethan-1-one)-*O,O*-dipyridin-2-ylmethyldioxime (**4**)


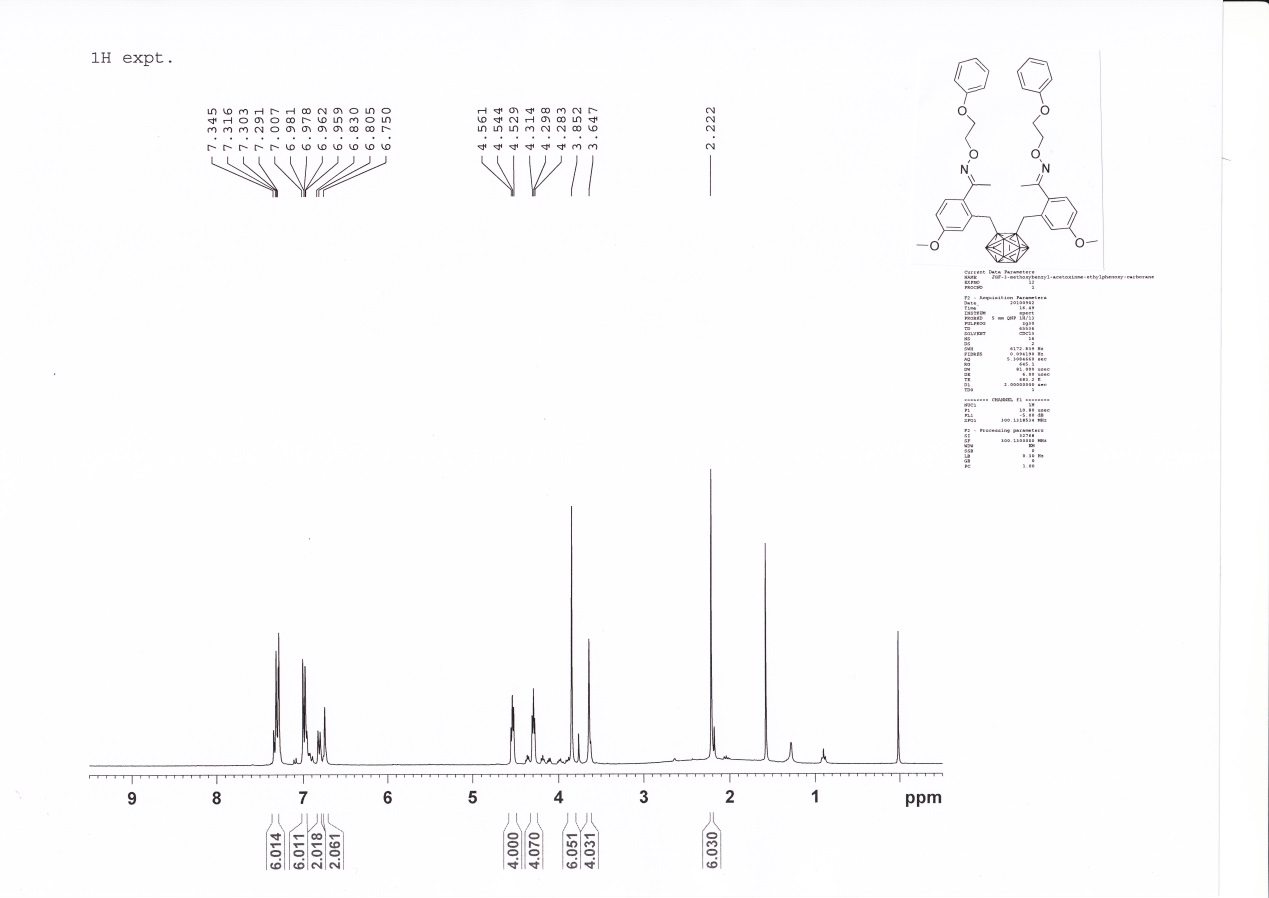


Additional file 1: FigS5. ^1^H-NMR (1*Z,*1*'Z*)-1,1'-(carboranyldimethyl)-bis(2-methoxy-4,1-phenylene-ethan-1-one)-*O,O*-di(2-phenoxyethyl)dioxime (**5**)


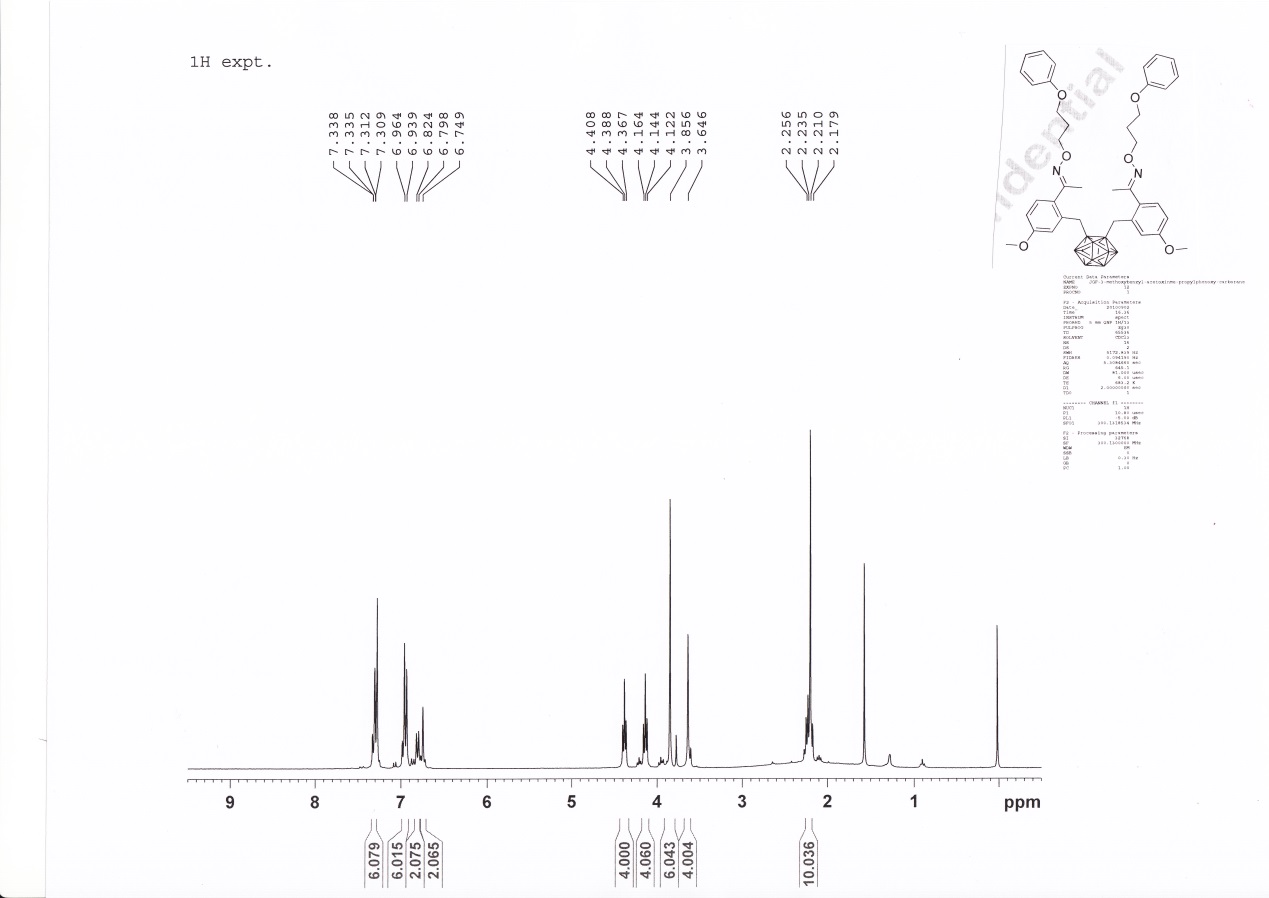
Additional file 1: FigS6. ^1^H-NMR (1*Z,*1*'Z*)-1,1'-(carboranyldimethyl)-bis(2-methoxy-4,1-phenylene-ethan-1-one)-*O,O*-di(3-phenoxypropyl)dioxime (**6**)


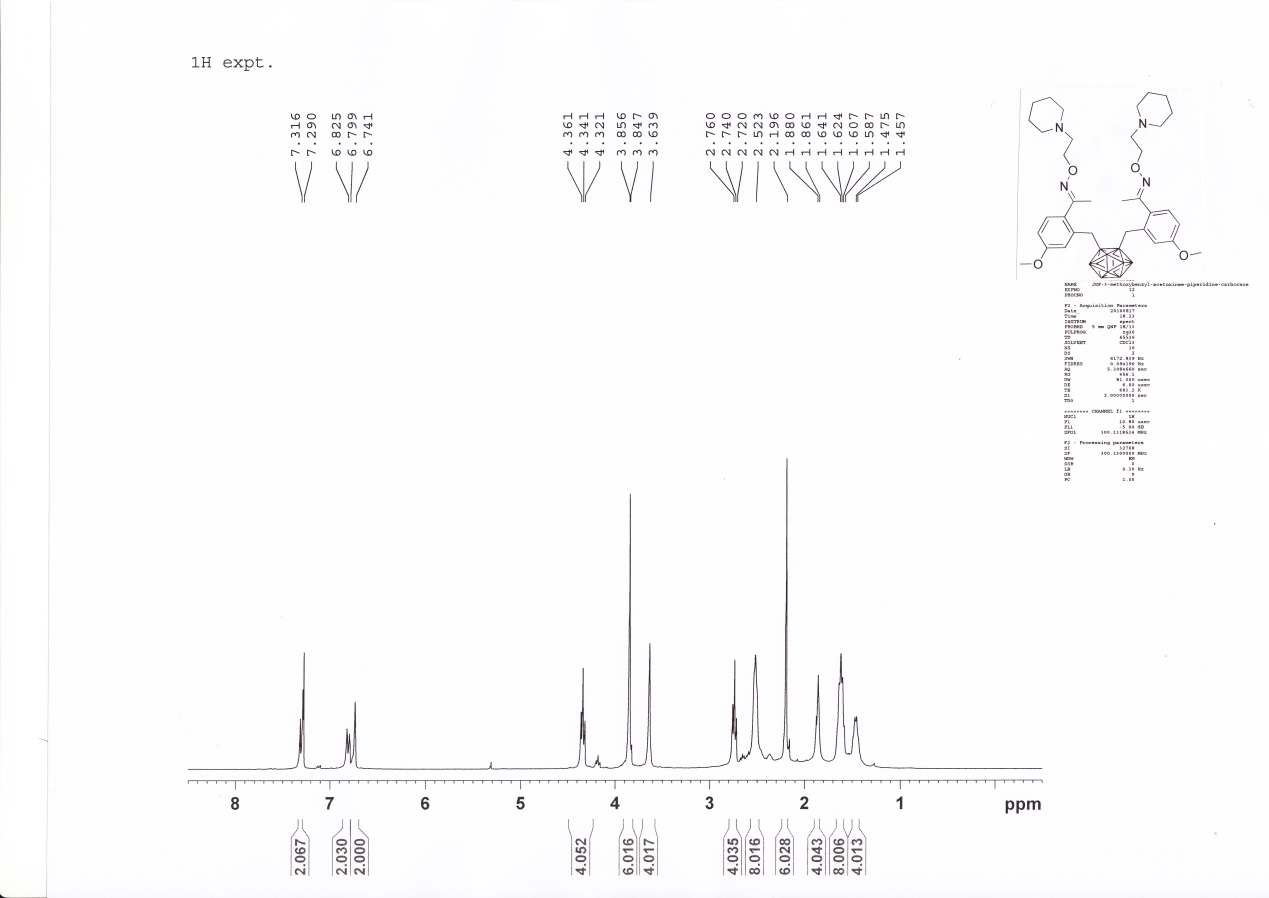


Additional file 1: FigS7. ^1^H-NMR (1*Z,*1*'Z*)-1,1'-(carboranyldimethyl)-bis(2-methoxy-4,1-phenylene-ethan-1-one)-*O,O*-di(2-piperidin-1-ylethyl)dioxime (**7**)


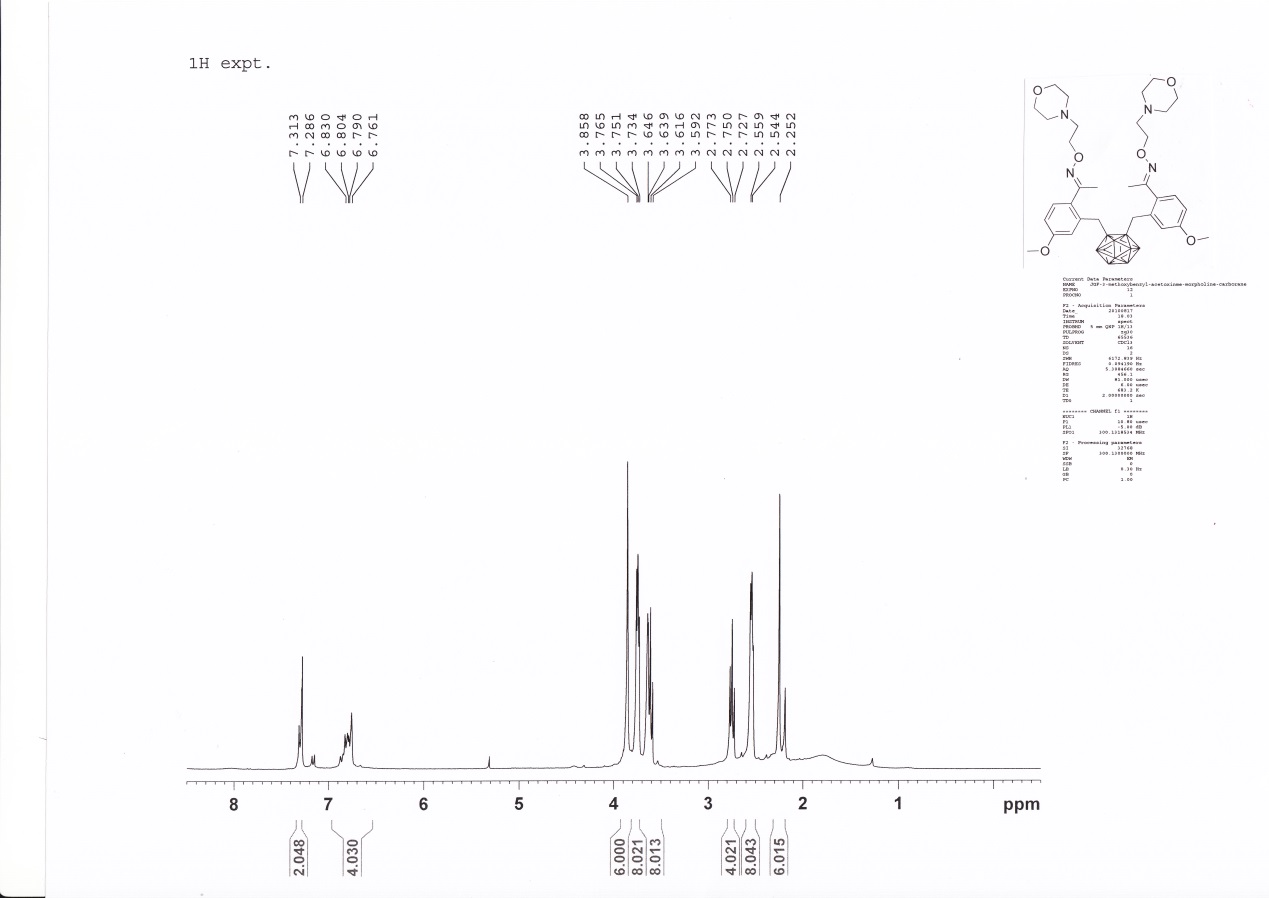
Additional file 1: FigS8. ^1^H-NMR (1*Z,*1*'Z)*-1,1'-(carboranyldimethyl)-bis(2-methoxy-4,1-phenylene-ethan-1-one)-*O,O*-di(2-morpholinoethyl)dioxime (**8**)
